# Patients prefer ChatGPT to institutional websites for questions on radiation-based imaging exams: international mixed-methods study

**DOI:** 10.1093/radadv/umag033

**Published:** 2026-07-23

**Authors:** Sofyan Jankowski, Charbel Mourad, Marie Nowak, Jonas Richiardi, Wendy Brito Rodriguez, Florian Poncet, Marianna Gulizia, Stephanie de Labouchere, Emily Harkness, David Rotzinger, Francesco Ria, Chiara Pozzessere

**Affiliations:** Department of Diagnostic and Interventional Radiology, Lausanne University Hospital (CHUV), Lausanne, 1011, Switzerland; Faculty of Biology and Medicine (FBM), University of Lausanne (UNIL), Lausanne, 1011, Switzerland; Department of Diagnostic and Interventional Radiology, Lebanese Hospital Geitaoui—UMC, Achrafieh, Geitaoui, 1100, Lebanon; Institute of Radiation Physics (IRA), University of Lausanne (UNIL), Lausanne, 1011, Switzerland; Department of Diagnostic and Interventional Radiology, Lausanne University Hospital (CHUV), Lausanne, 1011, Switzerland; CIBM Center for Biomedical Imaging, Lausanne, 1011, Switzerland; Department of Diagnostic and Interventional Radiology, Lausanne University Hospital (CHUV), Lausanne, 1011, Switzerland; Department of Diagnostic and Interventional Radiology, Lausanne University Hospital (CHUV), Lausanne, 1011, Switzerland; Department of Diagnostic and Interventional Radiology, Lausanne University Hospital (CHUV), Lausanne, 1011, Switzerland; Department of Radiologic Medical Imaging Technology, School of Health Sciences (HESAV), University of Applied Sciences and Arts Western Switzerland (HES-SO), Delémont, 1011, Switzerland; Clinical Imaging Physics Group, Department of Radiology, Duke University Medical Center, Durham, NC 27710, United States; Department of Diagnostic and Interventional Radiology, Lausanne University Hospital (CHUV), Lausanne, 1011, Switzerland; Faculty of Biology and Medicine (FBM), University of Lausanne (UNIL), Lausanne, 1011, Switzerland; Clinical Imaging Physics Group, Department of Radiology, Duke University Medical Center, Durham, NC 27710, United States; Carl E. Ravin Advanced Imaging Laboratories, Department of Radiology, Duke University Medical Center, Durham, NC 27710, United States; Center for Virtual Imaging Trials, Department of Radiology, Duke University Medical Center, Durham, NC 27710, United States; Department of Diagnostic and Interventional Radiology, Lausanne University Hospital (CHUV), Lausanne, 1011, Switzerland; Faculty of Biology and Medicine (FBM), University of Lausanne (UNIL), Lausanne, 1011, Switzerland

**Keywords:** radiation risk communication, ionizing radiation, artificial intelligence, patient education, patient satisfaction

## Abstract

**Background:**

Radiology-risk communication affects multiple clinical specialties that use ionizing radiation, and many patients seek related information online. Prior expert evaluations found comparable performance between ChatGPT-generated and radiology-risk answers from official institutions, but patient perspectives have not been assessed.

**Purpose:**

To assess patients’ perceptions of ChatGPT versus human-generated radiology-risk information.

**Methods and Materials:**

From December 2024 to March 2025, patients at 3 hospitals in the United States, Switzerland, and Lebanon were randomly assigned to 1 of 5 common radiology-risk questions. Participants, blinded to source, provided subjective ratings of both ChatGPT‑3.5 and human-generated institutional responses on 7-point Likert scales for satisfaction (primary outcome), comprehensibility, trust, and reassurance. Quantitative comparisons were performed with Inverse Normalizing Transformation, and free-text comments were analyzed using thematic coding.

**Results:**

A total of 328 patients participated (34% aged 18-39 years, 33% aged 40-59 years, 31% aged 60-79 years, 3% aged ≥80 years; 188 female). ChatGPT responses were rated significantly higher than human responses for satisfaction (0.70-point advantage; *P* < .001), comprehensibility (0.27 points; *P* < .01), trust (0.65 points; *P* < .001), and reassurance (0.51 points; *P* < .001). Findings converged with qualitative written comments (r = 0.91, *P* < .05), in which ChatGPT attracted 2.3× more positive comments while human-generated responses received 1.7× more negative comments.

**Conclusions:**

Unlike experts, patients preferred ChatGPT-generated responses to institutional materials for radiology risk questions. This divergence highlights the need for patient-centered communication and suggests that large language model-based styles, implemented with expert oversight, may improve the perceived clarity and trustworthiness of educational materials in medical specialties that use ionizing radiation.

**Summary** 

This multicenter study found that patients preferred ChatGPT-generated responses over human-written websites in satisfaction, comprehensibility, and trust regarding radiation-based imaging exams, suggesting that large language models may facilitate medical communication.

**Key Results** 

- Patients, blinded to authorship, rated ChatGPT’s responses to frequently asked questions about radiology risks 0.70 points higher (7-point Likert scale) than human-generated institutional website responses.
- Qualitative findings indicated ChatGPT attracted 2.3× more positive comments, whereas human-generated responses received 1.7× more negative comments.
- Human-generated responses were often described as “superficial” or “impersonal,” whereas ChatGPT text, though often described as “too long,” received spontaneous praise for “trustworthiness” and “satisfaction.”

## Introduction

Digital resources, including the Internet and, more recently, AI-based tools, have become important sources of health information for patients.^1^ Although some individuals remain skeptical of online health content,^2^ patients increasingly use web-based platforms for health care information search and self-diagnosis.^3^ This trend increased with the growing popularity of large language models (LLMs) and chatbots (eg, ChatGPT).

Communication about ionizing radiation is a useful test case for patient-facing AI. Questions about radiation exposure risks arise across multiple specialties, including radiology, nuclear medicine, interventional procedures guided by fluoroscopy, and radiation therapy. Although clinicians and institutions provide educational materials, patients may still experience uncertainty or concern and may turn to online sources for additional explanations.

Prior work on radiation-protection communication suggests that ChatGPT can improve readability and, in expert-based evaluations, perform comparably to institutional materials, although some responses may be incomplete or potentially misleading.^4^ Similar evaluations in other clinical contexts have reported that LLM outputs may be accurate and satisfactory, but concerns remain regarding reliability and perceived trustworthiness.^5^^,^^6^

However, most of assessments of LLMs’ clinical application rely on medical expert evaluation rather than real-world patient perspectives, leaving their viewpoints underrepresented in the literature.^7^^,^^8^ Patient-centered evaluation of material intended for patients is needed to inform how AI-generated content should be designed and improved over time.^9^ Therefore, the aim of this international multicentric study was to assess patients’ perceptions of ChatGPT versus human-generated radiology risk information in terms of satisfaction, comprehensibility, reassurance, and trust.

## Materials and methods

The study was conducted at 3 institutions: Lausanne University Hospital (Switzerland), Duke University Hospital (United States), and Lebanese Hospital Geitaoui (Lebanon). It was determined to be exempt from ethics approval by the legal services and/or ethical committees at Lausanne University Hospital (Exemption number Pro00117185), Duke University and was approved by the Lebanese Hospital Geitaoui Institutional Review Board (Decision 2024-IRB-044). No external funding was received.

This multicenter, blinded, within-subject paired design study evaluated subjective patient perceptions of ChatGPT versus human-generated responses to radiology risk FAQs from official institutional websites. The FAQs and responses were adapted from a prior expert-based study,^4^ from which 5 questions (Q1–Q5) were selected to cover diverse topics and message types (Table 1).

**Table 1:** Frequently asked patients’ questions, and responses from humans and ChatGPT.

**Question** *(item number in the previous expert-based study)*	Authorship of question and human response	Human response	ChatGPT response
Q1. What are the possible effects of radiation exposure from interventional procedures? *(Q4 in the expert-based study)*	https://www.iaea.org/resources/rpop/patients-and-public/interventional-procedures#3	The typical dose of an interventional procedure brings a 1 in 10 000 risk of skin injury and a small increased probability of cancer in future years. In most situations, skin injuries can be prevented by using the proper technique. An interventional procedure performed under X ray fluoroscopy guidance often replaces a more invasive surgical procedure.	While these procedures can be highly beneficial in diagnosing and treating various conditions, they also expose patients to ionizing radiation, which can have both short-term and long-term effects. Some possible effects of radiation exposure from interventional procedures include: Skin reactions: In some cases, radiation exposure can lead to skin reactions, such as redness, swelling, and, in severe cases, skin burns. Radiation sickness: High doses of radiation exposure can cause radiation sickness, leading to symptoms like nausea, vomiting, fatigue, and diarrhea. Cataracts: Repeated exposure to radiation may increase the risk of developing cataracts, clouding of the eye’s lens, leading to vision impairment. Infertility: Radiation exposure to the reproductive organs can potentially cause temporary or permanent infertility. Increased risk of cancer: Ionizing radiation is known to increase the risk of developing certain types of cancer, especially when received in high doses or over prolonged periods. However, the risk is generally considered to be low with standard medical imaging procedures. Radiation-induced tumors: Extremely high doses of radiation can, in rare cases, lead to the development of radiation-induced tumors in the area exposed to radiation. It’s important to note that the risk of experiencing adverse effects from radiation exposure during interventional procedures is typically outweighed by the potential benefits of accurate diagnosis and treatment. Health care professionals take precautions to minimize radiation exposure during procedures, such as using the lowest possible radiation dose necessary and employing shielding techniques to protect surrounding tissues and organs. If you are scheduled for an interventional procedure that involves radiation, it’s essential to discuss any concerns you may have with your health care provider. They can explain the specific risks and benefits of the procedure and address any questions you might have. Additionally, make sure to inform your health care provider of any existing medical conditions, allergies, or previous radiation exposures to ensure your safety during the procedure.
Q2. Hello. I am 36 years old now and have had undiagnosed illness for years. I have had a few CT scans, 3 nuclear stress tests, a few x-rays, ultrasounds, MRIs, and a combined CT abdomen/pelvis/chest (300 mGycm). I’m worried about radiation exposure. Does this sound a lot to you and can you please break the exposure levels down to me in layman’s terms? *(Q7 in the expert-based study)*	https://www.medicalradiationinfo.org/ask-the-experts/all-questions/	Not all imaging procedures use “ionizing” radiation, which is the type of radiation that has the potential to cause harm when received in high amounts. The ultrasound and MRI procedures you’ve had are examples of nonionizing procedures, so there is no concern about radiation effects. Your CT scans, nuclear scans, and radiographs (x-rays) used fairly small amounts of ionizing radiation to produce images. The radiation dose level that you provided for your CT scan of the chest/abdomen/pelvis (300 mGycm) is extremely low compared to national averages for that (very common) imaging examination. The information obtained from medically necessary imaging like CT scanning, nuclear imaging, and x-rays is often very beneficial to medical diagnosis and patient outcomes. The small amount of ionizing radiation used in medical imaging has not been scientifically proven to cause any harm, and if there is any long-term risk, then it is believed to be extremely small. If radiation exposures are separated by weeks or months, then the body’s innate ability to repair DNA damage will go into action, further reducing the small theoretical risk. There is no limit on the amount of medical imaging a patient can receive. Each imaging exam is ordered based on the patient’s situation at that particular time, without regard to their radiation exposure history. This is good, because it means that a patient should not be denied a potentially life-preserving imaging examination just because they may have had prior imaging. With modern imaging equipment, the amount of radiation used to produce images is customized for the patient’s body size and for the purpose of the examination.	I understand your concerns about radiation exposure. Let me break down the types of medical imaging you’ve had and provide some context on radiation levels in layman’s terms: X-rays: X-rays are a type of electromagnetic radiation used for imaging bones and some soft tissues. They are relatively low-dose radiation exams, and the exposure from each X-ray is generally considered safe. The risk associated with a single X-ray is minimal. CT scans: CT scans involve a series of X-ray images taken from different angles to create cross-sectional images of the body. CT scans do expose you to a higher dose of radiation compared to conventional X-rays, but they are still considered safe for medical purposes. The exposure from a single CT scan is generally not harmful, but repeated CT scans can accumulate radiation exposure over time. MRIs: MRIs use strong magnetic fields and radio waves to create detailed images of the body’s internal structures. Unlike X-rays and CT scans, MRIs do not use ionizing radiation, so there is no radiation exposure associated with this imaging method. Nuclear stress tests: Nuclear stress tests involve injecting a small amount of radioactive tracer into your bloodstream to evaluate blood flow to the heart. The radiation dose from nuclear stress tests is higher than conventional X-rays, but it is still considered safe for diagnostic purposes. Radiation exposure is typically measured in units called “milligray” (mGy) or “millisievert” (mSv). The effective dose of radiation takes into account the type of radiation and the sensitivity of different organs to radiation exposure. For example: A typical chest X-ray exposes you to about 0.1 mSv. A CT scan of the abdomen and pelvis may expose you to around 10 mSv. A nuclear stress test can expose you to between 5 to 15 mSv. It’s essential to note that the risk of developing radiation-induced health effects increases with higher doses and cumulative exposure over time. However, the doses you receive during medical imaging are generally kept within safe limits, and the benefits of these tests in diagnosing and managing medical conditions often outweigh the risks.
Q3. How long should I wait to try to become pregnant after x-ray exams that have exposed my ovaries or my husband’s testes? *(Q8 in the expert-based study)*	https://www.radiationanswers.org/radiation-questions-answers/radiation-and-pregnancy.html	There is no evidence of effects on the ovaries or sperm at radiation doses used in diagnostic x-ray procedures. Radiation doses from routine medical x rays are too small to affect sterility or have an effect on a future pregnancy. Therefore, you do not need to wait after diagnostic x-ray examinations before trying to become pregnant.	The waiting period before attempting to become pregnant after x-ray exams that exposed the ovaries or testes can vary depending on the type of exam, the radiation dose received, and individual factors. It is best to consult with a health care professional, such as a radiologist or a reproductive specialist, who can provide personalized advice based on your specific situation. They can review your medical history, the details of the x-ray examinations, and any other relevant factors to provide you with appropriate guidance on when it is safe to try to conceive after the exposure. They may also recommend follow-up tests or monitoring to ensure your reproductive health is not adversely affected. In general, the International Commission on Radiological Protection (ICRP) recommends that women avoid becoming pregnant for a period of time after exposure to significant radiation doses. This period is typically around 1 to 3 months for women and about two to three months for men.
Q4. Do patients need to cease breastfeeding after CT with iodine contrast? *(Q10 in the expert-based study)*	https://medicine.yale.edu/diagnosticradiology/patientcare/policies/breastfeeding/	For all IV iodinated contrast and gadolinium, American College of Radiology (ACR) guidelines state that contrast administration to the mother is considered safe for both the baby and nursing mother. Mothers who are breastfeeding should be given the opportunity to make an informed decision as to whether to continue or temporarily abstain from breastfeeding after receiving IV contrast. If the mother remains concerned about any potential ill effects to the infant, she may abstain from breast-feeding for 24 hours with active expression and discarding of breast milk from both breasts during that period.	In general, after receiving iodine contrast for a CT scan, the recommendation is typically to temporarily suspend breastfeeding for a short period. Iodine contrast agents used in medical imaging can pass into breast milk, and there may be potential risks to the infant. The specific guidelines for how long to stop breastfeeding after a CT with iodine contrast can vary depending on the type of contrast used and the recommendations of the radiologist or health care provider. Generally, breastfeeding can be resumed after a certain waiting period, during which the iodine contrast is cleared from the body. If you are a breastfeeding parent and have been scheduled for a CT with iodine contrast, it is crucial to discuss your specific situation with your healthcare provider. They will be able to provide you with the most accurate and up-to-date information and guidance regarding breastfeeding and medical imaging procedures.
Q5. If radiation is harmful to humans, how can it also be used to treat cancer? *(Q12 in the expert-based study)*	https://www.medicalradiationinfo.org/ask-the-experts/all-questions/	Radiation can damage cells in the body. Healthy, normal cells are capable of repairing themselves following exposure to radiation. However, cancerous cells have lost this ability. When radiation is delivered to a part of the body containing a tumour, the cancerous cells are killed but the noncancerous cells survive.	Radiation can indeed be harmful to humans, but it can also be used as an effective treatment for cancer. The key to its successful use lies in carefully controlling the dose and targeting the radiation to specific areas of the body where cancer cells are present. Radiation therapy, also known as radiotherapy, is a medical treatment that uses high doses of radiation to kill or damage cancer cells. It works by damaging the DNA inside the cancer cells, preventing them from growing and dividing further. While radiation can also affect normal healthy cells, the goal of radiation therapy is to maximize the damage to cancer cells while minimizing harm to healthy tissues. Here’s how radiation therapy achieves this balance: Precise targeting: Modern radiation therapy techniques, such as intensity-modulated radiation therapy (IMRT) and image-guided radiation therapy (IGRT), allow for highly accurate and precise targeting of the tumour. This reduces exposure to surrounding healthy tissues, which helps minimize damage to non-cancerous cells. Fractionation: Radiation treatment is usually delivered in multiple sessions or fractions over several days or weeks. By dividing the total radiation dose into fractions, the healthy cells have time to repair themselves between treatments, while cancer cells are less likely to recover. Shielding and blocking: Special shields and blocks may be used during radiation therapy to protect nearby organs and tissues that are sensitive to radiation. Advanced planning and imaging: Before treatment, detailed planning is done using advanced imaging techniques to determine the exact location and size of the tumor, as well as nearby critical structures. This enables the medical team to design a treatment plan that delivers the radiation precisely to the tumor while sparing healthy tissues. Radiosensitivity of cancer cells: Cancer cells are generally more sensitive to radiation than normal cells, making them more susceptible to damage and death at the same radiation dose that may only cause temporary effects on healthy cells. While radiation therapy is a valuable tool in cancer treatment, it does have potential side effects, which can vary depending on the area being treated and the overall health of the patient. Common side effects include fatigue, skin changes, and mild inflammation of the treated area. However, most side effects are temporary and resolve after treatment is completed. It’s crucial for patients considering radiation therapy to discuss the potential risks and benefits with their oncologist to make informed decisions about their cancer treatment plan. Every patient’s situation is unique, and the decision to use radiation therapy will depend on factors such as cancer type, stage, location, and the patient’s overall health.

Adapted and reprinted, with permission, from reference.^4^

### Participant enrollment

Between December 2024 and March 2025, consecutive adult patients (≥18 years old) capable of providing informed consent and able to read who presented in the radiology waiting rooms of the three institutions were invited to participate. Recruitment was conducted by staff (radiologists, radiographers, medical physicists, receptionists) or via self-enrolment using QR codes on posters displayed in waiting areas.

Each participant who volunteered to participate, regardless of their own examination scheduled, was randomly assigned to 1 of 5 question sets (Q1-Q5) in a 1:1:1:1:1 ratio and blindly evaluated both ChatGPT and human responses, labeled “Author A” or “Author B.” For paper forms, the study team (C.M., F.R., C.P., S.J., E.H.) shuffled the 5 versions; the author order was fixed, with human responses first for Q1-Q3 and ChatGPT first for Q4-Q5. For online forms, patients were randomly assigned to a version with randomized response order.

Participants also provided demographic and background information including sex, country, age, education, occupation, number of imaging examinations in the previous 12 months and lifetime, prior use of AI chatbots for medical information, and prior searches for radiation protection information in medical imaging.

This study focuses on subjective patient assessments and does not include evaluation of clinical content accuracy, which was addressed in a prior study.^4^

### Quantitative outcomes

The participants were invited to rate ChatGPT and human-generated responses using a 7-point Likert scale (1 = no; 7 = yes). Four outcomes based on the QUEST framework principles for LLM evaluation in health care were included:^10^

- Satisfaction: “I am satisfied with the answer,” as the primary outcome.
- Comprehensibility: “The text is understandable.”
- Trust: “I trust the information presented in the text.”
- Reassurance: “This answer is reassuring.”

Moreover, the participants were asked to identify whether an answer was generated by AI (*“The answer was generated by AI”*).

A sample questionnaire form is shown in Figure S1.

### Qualitative outcomes

Patients could also add optional free-text comments through open-ended prompts asking how Author A’s and Author B’s responses could be improved, and whether they had any additional questions regarding radiation exposure, safety, or the potential harmful effects of radiation in medical imaging. Comments in French and Arabic were translated into English by the native-speaking authors, with assistance from Microsoft Copilot Excel.

### Statistical analysis

Sample size was calculated to detect the author effect in the primary outcome using paired *t*-test formulas. Based on a previous study,^4^ we assumed a standard deviation of paired differences of 2.97 on a 7-point Likert scale and an expected mean difference of 0.5. With 2-sided α = 0.05 and 80% power, 279 participants were required.

Descriptive statistics included Likert scale means, standard deviations, medians, interquartile ranges, and minimum/maximum values of Likert scale scores. Only questionnaires with complete demographic and Likert scale data were included in the analysis. Outcomes were analyzed using linear mixed-effects models with inverse normal transformation (INT). Model selection compared the base model (author×question) with the covariate-adjusted model (age, sex, country) using likelihood ratio tests and Akaike information criterion. A random intercept accounted for paired author ratings within subjects. Effect sizes were calculated as partial η^2^ for F-tests of main effects and interactions and as Cohen’s d for pairwise contrasts. To facilitate interpretation, contrasts from the INT model were back-transformed to the original 1 to 7 Likert scale by multiplying by the outcome’s standard deviation; this value serves solely as a descriptive aid to contextualize effect magnitude.

For the primary outcome (satisfaction), the main effect of author type represented our a priori hypothesis and was tested without correction at α = 0.05. For other outcomes (comprehensibility, trust, reassurance, AI identification), author effect *P* values were adjusted using Benjamini-Hochberg false discovery rate (Q = 0.05). Post hoc comparisons for the author×question interaction were adjusted using Holm-Bonferroni sequential correction. Pairwise comparisons used estimated marginal means with Kenward–Roger degrees of freedom approximation.

A nonparametric sensitivity analysis using aligned rank transformation ANOVA was performed. All analyses were conducted in R (version 4.3.1) using lmerTest, emmeans, pwr, and ARTool packages. The R code for the statistical analyses is available on reasonable request from the corresponding author.

### Qualitative outcomes analysis

A hybrid inductive-deductive content analysis was employed to allow themes to emerge from the data (inductive phase) while ensuring structured alignment with predefined Likert-scale outcomes (deductive phase) for mixed-methods integration (Supplemental Materials Methods).

### Mixed methods integration

Counts of positive and negative *meaning units* extracted from participants’ optional free-text comments were tallied as $n_{\text{positive}}$ and $n_{\text{negative}}$,

The Valence Index, summarizing the balance of positive and negative *meaning units*, was calculated as:


$$\text{Valence}\;\text{Index}=\frac{n_{\text{positive}}-n_{\text{negative}}}{n_{\text{positive}}+n_{\text{negative}}}$$

yielding values between -1.0 (exclusively negative) and +1.0 (exclusively positive sentiment).

The Valence Differential expressed the qualitative contrast between authors (positive = preference for ChatGPT; negative = preference for Human).


$$\text{Valence}\;\text{Differential}={\text{Valence}\;\text{Index}}_{\text{ChatGPT}}-{\text{Valence}\;\text{Index}}_{\text{Human}}$$

Convergence between qualitative sentiment (valence differential) and quantitative outcomes (Cohen’s d) was evaluated using Pearson correlations.

## Results

### Participant characteristics

**Figure 1:**
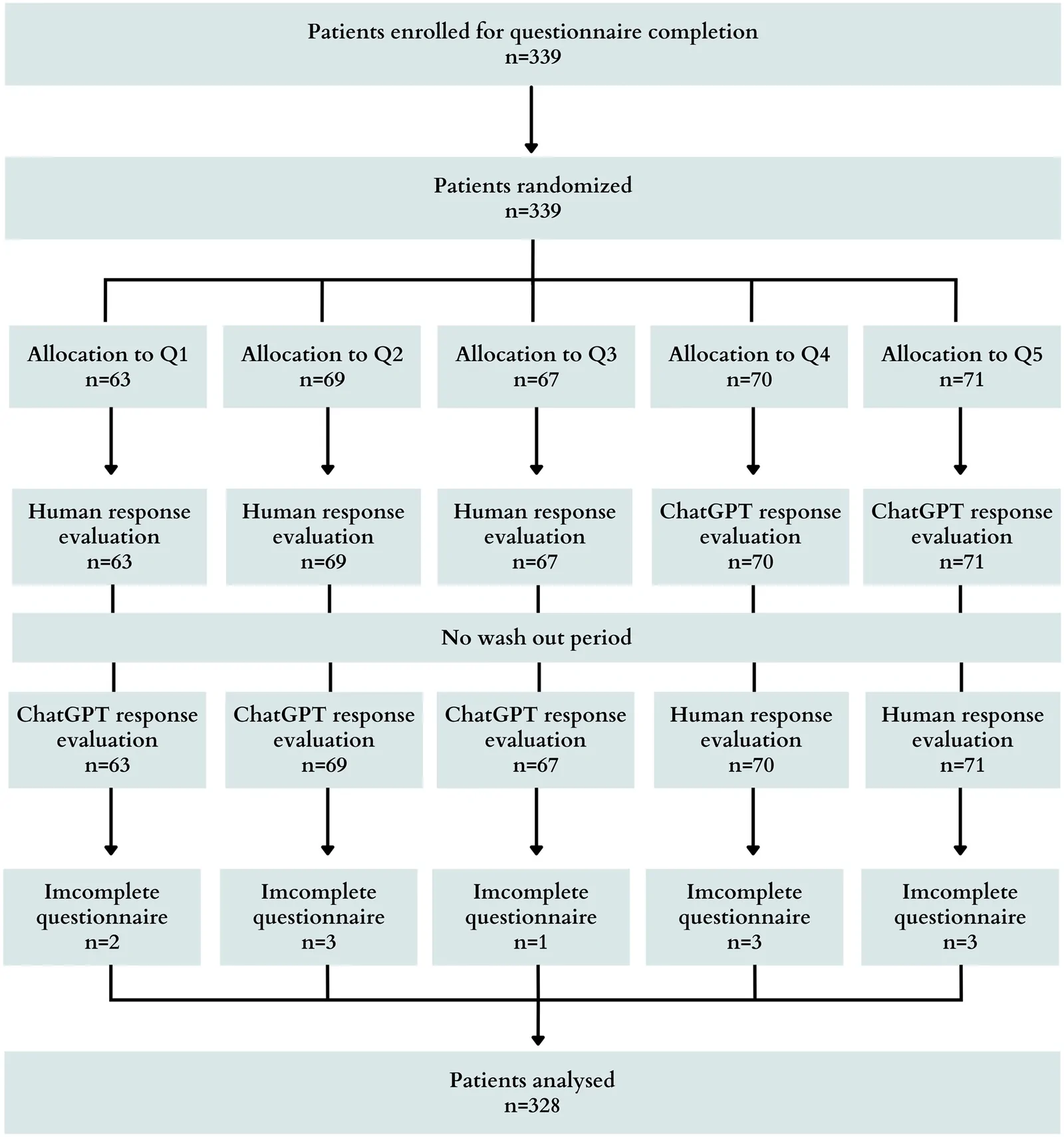
Flow of patients through recruitment, randomization, and analysis.

A total of 328 forms were fully completed and subsequently included in the final analysis (Figure 1). Of these patients, 188 (57%) were females, 129 (39%) were from Switzerland, 101 (31%) from the United States, and 98 (30%) from Lebanon. Five percent of respondents had never heard of LLMs, whereas up to 29% had previously used them as a source of medical information. Up to 57% of respondents expressed interest in a dedicated Chatbot for radiation protection-related inquiries. Details about all patients’ sociodemographic information are presented in Table 2.

**Table 2:** Baseline characteristics by randomized question group.

	Q1	Q2	Q3	Q4	Q5	Overall
	(*N* = 61)	(*N* = 66)	(*N* = 66)	(*N* = 67)	(*N* = 68)	(*N* = 328)
**Form Format**						
Online	9 (14.8%)	7 (10.6%)	12 (18.2%)	11 (16.4%)	11 (16.2%)	50 (15.2%)
Paper	52 (85.2%)	59 (89.4%)	54 (81.8%)	56 (83.6%)	57 (83.8%)	278 (84.8%)
**Language**						
Arabic	7 (11.5%)	11 (16.7%)	13 (19.7%)	12 (17.9%)	8 (11.8%)	51 (15.5%)
English	23 (37.7%)	26 (39.4%)	26 (39.4%)	28 (41.8%)	32 (47.1%)	135 (41.2%)
French	31 (50.8%)	29 (43.9%)	27 (40.9%)	27 (40.3%)	28 (41.2%)	142 (43.3%)
**Country**						
Lebanon	13 (21.3%)	22 (33.3%)	21 (31.8%)	19 (28.4%)	23 (33.8%)	98 (29.9%)
Switzerland	29 (47.5%)	25 (37.9%)	27 (40.9%)	25 (37.3%)	23 (33.8%)	129 (39.3%)
United States	19 (31.1%)	19 (28.8%)	18 (27.3%)	23 (34.3%)	22 (32.4%)	101 (30.8%)
**Age group**						
18 to 39	18 (29.5%)	25 (37.9%)	21 (31.8%)	20 (29.9%)	26 (38.2%)	110 (33.5%)
40 to 59	21 (34.4%)	21 (31.8%)	21 (31.8%)	25 (37.3%)	20 (29.4%)	108 (32.9%)
60 to 79	19 (31.1%)	18 (27.3%)	24 (36.4%)	18 (26.9%)	21 (30.9%)	100 (30.5%)
80 +	3 (4.9%)	2 (3.0%)	0 (0%)	4 (6.0%)	1 (1.5%)	10 (3.0%)
**Sex**						
Female	28 (45.9%)	44 (66.7%)	34 (51.5%)	37 (55.2%)	45 (66.2%)	188 (57.3%)
Male	33 (54.1%)	22 (33.3%)	32 (48.5%)	30 (44.8%)	23 (33.8%)	140 (42.7%)
**Education level**						
Primary school	1 (1.6%)	3 (4.5%)	0 (0%)	3 (4.5%)	0 (0%)	7 (2.1%)
Middle school	4 (6.6%)	4 (6.1%)	0 (0%)	2 (3.0%)	1 (1.5%)	11 (3.4%)
High school	13 (21.3%)	17 (25.8%)	22 (33.3%)	20 (29.9%)	18 (26.5%)	90 (27.4%)
Post high school	34 (55.7%)	31 (47.0%)	27 (40.9%)	25 (37.3%)	30 (44.1%)	147 (44.8%)
Graduate/postgraduate	9 (14.8%)	11 (16.7%)	17 (25.8%)	17 (25.4%)	19 (27.9%)	73 (22.3%)
**Occupation**						
Student	5 (8.2%)	8 (12.1%)	3 (4.5%)	4 (6.0%)	9 (13.2%)	29 (8.8%)
Unemployed	1 (1.6%)	2 (3.0%)	3 (4.5%)	9 (13.4%)	0 (0%)	15 (4.6%)
Employed	28 (45.9%)	34 (51.5%)	28 (42.4%)	27 (40.3%)	30 (44.1%)	147 (44.8%)
Freelance	2 (3.3%)	5 (7.6%)	6 (9.1%)	7 (10.4%)	5 (7.4%)	25 (7.6%)
Housewife/househusband	3 (4.9%)	5 (7.6%)	6 (9.1%)	4 (6.0%)	7 (10.3%)	25 (7.6%)
Retired	22 (36.1%)	12 (18.2%)	20 (30.3%)	16 (23.9%)	17 (25.0%)	87 (26.5%)
**Imaging tests (past 12 months)**						
0 examinations	9 (14.8%)	13 (19.7%)	14 (21.2%)	14 (20.9%)	13 (19.1%)	63 (19.2%)
1 examination	21 (34.4%)	24 (36.4%)	27 (40.9%)	23 (34.3%)	29 (42.6%)	124 (37.8%)
2-5 examinations	24 (39.3%)	22 (33.3%)	17 (25.8%)	16 (23.9%)	20 (29.4%)	99 (30.2%)
6-10 examinations	7 (11.5%)	6 (9.1%)	5 (7.6%)	13 (19.4%)	2 (2.9%)	33 (10.1%)
More than 10 examinations	0 (0%)	1 (1.5%)	3 (4.5%)	1 (1.5%)	4 (5.9%)	9 (2.7%)
**Imaging tests (lifetime)**						
0 examinations	2 (3.3%)	5 (7.6%)	4 (6.1%)	2 (3.0%)	3 (4.4%)	16 (4.9%)
1 examination	10 (16.4%)	7 (10.6%)	9 (13.6%)	3 (4.5%)	10 (14.7%)	39 (11.9%)
2-5 examinations	16 (26.2%)	17 (25.8%)	14 (21.2%)	15 (22.4%)	20 (29.4%)	82 (25.0%)
6-10 examinations	9 (14.8%)	17 (25.8%)	6 (9.1%)	13 (19.4%)	10 (14.7%)	55 (16.8%)
More than 10 examinations	24 (39.3%)	20 (30.3%)	33 (50.0%)	34 (50.7%)	25 (36.8%)	136 (41.5%)
**Have you ever used AI chatbots (ie, ChatGPT) for seeking medical information?**						
I don’t know what it is	6 (9.8%)	3 (4.5%)	3 (4.5%)	4 (6.0%)	1 (1.5%)	17 (5.2%)
No	30 (49.2%)	41 (62.1%)	32 (48.5%)	31 (46.3%)	29 (42.6%)	163 (49.7%)
No but I use it for other purposes	9 (14.8%)	9 (13.6%)	11 (16.7%)	10 (14.9%)	14 (20.6%)	53 (16.2%)
Yes	16 (26.2%)	13 (19.7%)	20 (30.3%)	22 (32.8%)	24 (35.3%)	95 (29.0%)
**Have you ever sought information on radiation exposure, safety, and potential harmful effects of radiation in medical imaging?**						
No	41 (67.2%)	47 (71.2%)	38 (57.6%)	33 (49.3%)	42 (61.8%)	201 (61.3%)
Yes, both	6 (9.8%)	5 (7.6%)	13 (19.7%)	19 (28.4%)	11 (16.2%)	54 (16.5%)
Yes, I asked health care professionals	7 (11.5%)	9 (13.6%)	12 (18.2%)	9 (13.4%)	7 (10.3%)	44 (13.4%)
Yes, on the internet	7 (11.5%)	5 (7.6%)	3 (4.5%)	6 (9.0%)	8 (11.8%)	29 (8.8%)
**Are you interested in a dedicated chatbot to ask for specific radiology questions including radiation exposure, safety, and potential harmful effects of radiation in medical imaging?**						
No	29 (47.5%)	30 (45.5%)	28 (42.4%)	25 (37.3%)	30 (44.1%)	142 (43.3%)
Yes, both	17 (27.9%)	11 (16.7%)	19 (28.8%)	27 (40.3%)	19 (27.9%)	93 (28.4%)
Yes, in the radiology waiting room	8 (13.1%)	15 (22.7%)	14 (21.2%)	8 (11.9%)	8 (11.8%)	53 (16.2%)
Yes, on the internet	7 (11.5%)	10 (15.2%)	5 (7.6%)	7 (10.4%)	11 (16.2%)	40 (12.2%)

### Quantitative analysis

**Figure 2:**
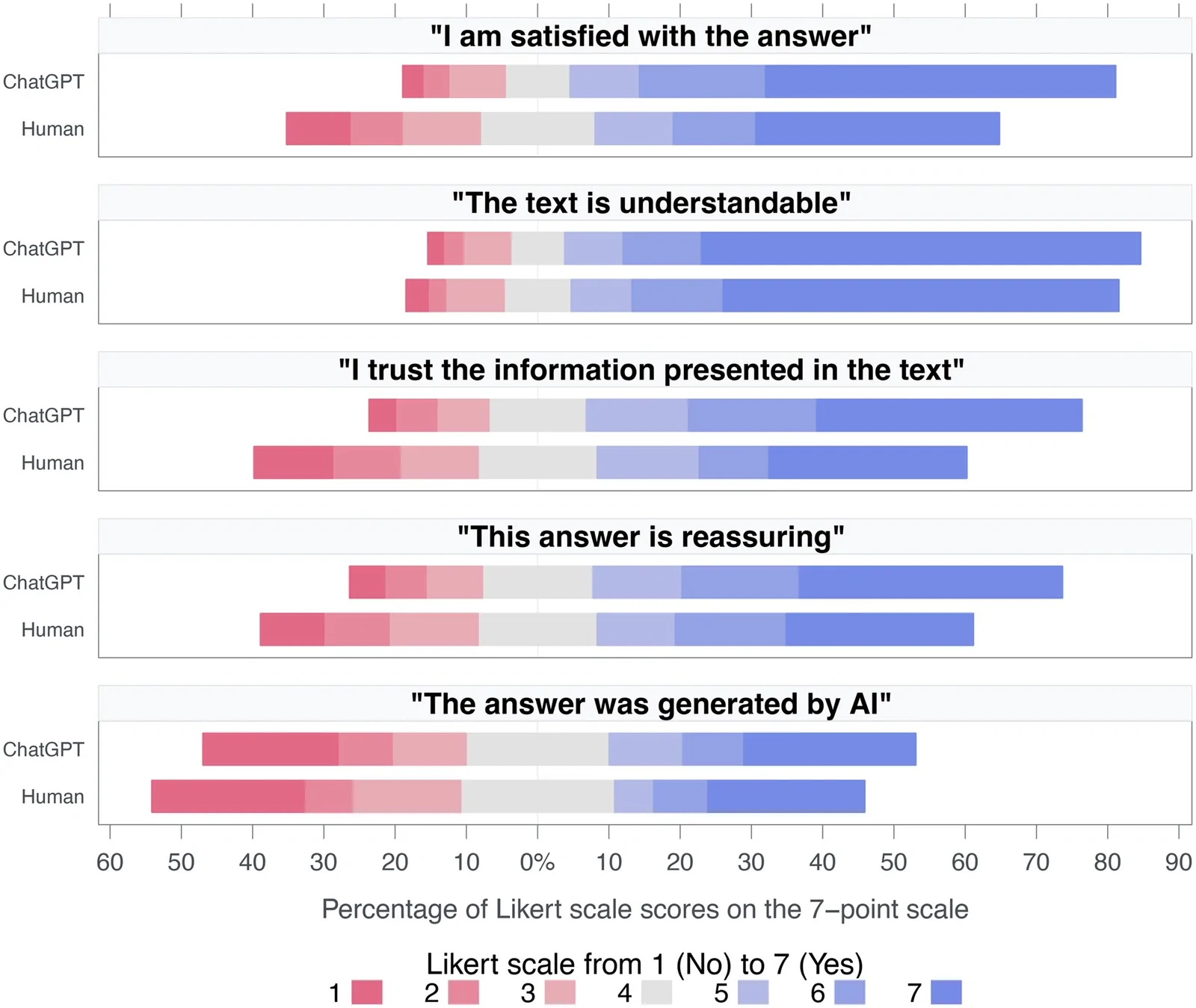
Likert plot of participants’ ratings for ChatGPT and human-generated responses. Positive ratings (shades of blue) were more frequent for ChatGPT responses, whereas negative ratings (shades of red) were more common for human-generated content. The figure shows the distribution of participants’ Likert scale ratings (1 = “No” to 7 = “Yes”) after evaluating both ChatGPT- and human-generated responses to radiology risk questions. Each bar represents the percentage of ratings from 1 to 7 across all participants evaluating 1 of 5 questions.

Patient evaluations were classified by outcome and question (Table 3), stratified by country (Table S1). Participants’ Likert scale ratings are shown in Figure 2.

**Table 3:** Means, standard deviations, medians, interquartile ranges (IQR), minimum and maximum scores for ChatGPT, and human-generated responses to patients’ questions, presented for each outcome.

Question		Q1	Q1	Q2	Q2	Q3	Q3	Q4	Q4	Q5	Q5	All questions	All questions	All questions
Outcome		ChatGPT	Human	ChatGPT	Human	ChatGPT	Human	ChatGPT	Human	ChatGPT	Human	ChatGPT	Human	Overall
**Satisfaction**	Mean ± SD	5.6 ± 1.8	4.7 ± 2.0	5.6 ± 1.6	5.5 ± 1.8	5.6 ± 2.1	4.8 ± 2.3	5.9 ± 1.5	4.7 ± 1.9	5.8 ± 1.4	4.5 ± 2.1	5.7 ± 1.7	4.8 ± 2.0	5.3 ± 1.9
	Median (IQR)	7.0 (3.0)	5.0 (4.0)	6.0 (2.8)	6.0 (2.8)	7.0 (2.8)	5.5 (4.0)	6.0 (2.0)	4.0 (2.5)	6.0 (2.0)	4.0 (4.0)	6.0 (2.0)	5.0 (4.0)	5.5 (3.0)
	Range [min; max]	[1; 7]	[1; 7]	[1; 7]	[1; 7]	[1; 7]	[1; 7]	[2; 7]	[1; 7]	[1; 7]	[1; 7]	[1; 7]	[1; 7]	[1; 7]
**Comprehensibility**	Mean ± SD	6.0 ± 1.6	5.6 ± 1.8	6.0 ± 1.6	6.0 ± 1.4	5.8 ± 1.9	6.1 ± 1.7	6.2 ± 1.3	5.5 ± 1.8	5.8 ± 1.7	5.8 ± 1.6	6.0 ± 1.6	5.8 ± 1.7	5.9 ± 1.7
	Median (IQR)	7.0 (2.0)	7.0 (3.0)	7.0 (1.0)	7.0 (2.0)	7.0 (2.0)	7.0 (1.0)	7.0 (1.0)	6.0 (3.0)	7.0 (2.2)	7.0 (2.2)	7.0 (2.0)	7.0 (2.0)	7.0 (2.0)
	Range [min; max]	[1; 7]	[1; 7]	[1; 7]	[1; 7]	[1; 7]	[1; 7]	[2; 7]	[1; 7]	[1; 7]	[1; 7]	[1; 7]	[1; 7]	[1; 7]
**Trustworthiness**	Mean ± SD	5.3 ± 1.9	4.8 ± 2.1	5.4 ± 1.6	5.1 ± 1.8	5.4 ± 2.0	4.2 ± 2.4	5.3 ± 1.6	4.2 ± 1.9	5.2 ± 1.7	4.4 ± 2.0	5.3 ± 1.8	4.5 ± 2.1	4.9 ± 1.9
	Median (IQR)	6.0 (3.0)	5.0 (4.0)	6.0 (3.0)	5.5 (3.0)	7.0 (3.0)	4.0 (5.0)	6.0 (3.0)	4.0 (3.0)	5.0 (3.0)	4.5 (3.0)	6.0 (3.0)	5.0 (4.0)	5.5 (3.5)
	Range [min; max]	[1; 7]	[1; 7]	[1; 7]	[1; 7]	[1; 7]	[1; 7]	[1; 7]	[1; 7]	[1; 7]	[1; 7]	[1; 7]	[1; 7]	[1; 7]
**Reassurance**	Mean ± SD	4.7 ± 2.0	4.2 ± 1.9	5.3 ± 1.8	5.6 ± 1.7	5.1 ± 2.1	4.5 ± 2.3	5.5 ± 1.6	4.5 ± 1.8	5.4 ± 1.6	4.3 ± 1.9	5.2 ± 1.8	4.6 ± 2.0	4.9 ± 1.9
	Median (IQR)	5.0 (4.0)	4.0 (3.0)	6.0 (3.0)	6.0 (2.0)	6.0 (4.0)	5.0 (4.0)	6.0 (3.0)	4.0 (2.0)	6.0 (3.0)	4.0 (3.0)	6.0 (3.0)	5.0 (4.0)	5.5 (3.5)
	Range [min; max]	[1; 7]	[1; 7]	[1; 7]	[1; 7]	[1; 7]	[1; 7]	[2; 7]	[1; 7]	[1; 7]	[1; 7]	[1; 7]	[1; 7]	[1; 7]
**All outcomes**	Mean ± SD	5.4 ± 0.5	4.8 ± 0.6	5.6 ± 0.3	5.5 ± 0.4	5.5 ± 0.3	4.9 ± 0.8	5.7 ± 0.4	4.7 ± 0.6	5.5 ± 0.3	4.7 ± 0.7	5.5 ± 0.3	5.0 ± 0.6	5.2 ± 0.5
	Median (IQR)	6.5 (1.2)	5.0 (0.8)	6.0 (0.2)	6.0 (0.4)	7.0 (0.2)	5.2 (1.1)	6.0 (0.2)	4.0 (0.5)	6.0 (0.5)	4.2 (1.1)	6.0 (0.2)	5.0 (0.5)	5.5 (0.4)
	Range [min; max]	[1; 7]	[1; 7]	[1; 7]	[1; 7]	[1; 7]	[1; 7]	[1; 7]	[1; 7]	[1; 7]	[1; 7]	[1; 7]	[1; 7]	[1; 7]
**AI—Identification**	Mean ± SD	4.4 ± 2.4	3.7 ± 2.4	4.1 ± 2.3	3.8 ± 2.2	3.9 ± 2.3	4.5 ± 2.1	3.7 ± 1.8	4.0 ± 2.0	4.7 ± 2.0	3.7 ± 2.1	4.2 ± 2.2	3.9 ± 2.2	4.1 ± 2.2
	Median (IQR)	5.0 (5.0)	3.0 (5.0)	4.0 (4.0)	3.5 (4.0)	4.0 (4.0)	4.0 (4.0)	4.0 (2.0)	4.0 (3.0)	5.0 (3.0)	4.0 (3.0)	4.0 (4.0)	4.0 (4.0)	4.0 (4.0)
	Range [min; max]	[1; 7]	[1; 7]	[1; 7]	[1; 7]	[1; 7]	[1; 7]	[1; 7]	[1; 7]	[1; 7]	[1; 7]	[1; 7]	[1; 7]	[1; 7]

Abbreviations: AI, artificial Intelligence; SD, standard deviation.

#### Primary outcome

Overall satisfaction scores favored ChatGPT-generated responses (Median = 6, IQR = 5-7) compared to human-generated responses (Median = 5, IQR = 3-7), the detailed score distributions are presented in Figure 2.

The inclusion of demographic covariates (age, sex, and country) significantly improved model fit over the base model (χ^2^ = 45.7, *df  *= 6, *P* < .001) and demonstrated superior fit indices (Akaike information criterion = 1617 vs. 1631)

##### Main effects

Author effect: A significant main effect of author emerged, F(1,323) = 46.1, *P* < .001, η^2^=0.12 indicating a moderate effect by conventional benchmarks(small: η^2^ = 0.01, moderate: η^2^ = 0.06, large: η^2^ = 0.14).^11^ We found a higher standardized INT ratings for ChatGPT than Human (estimated marginal means: GPT = 0.21, SE = 0.06; Human=-0.16, SE = 0.06; standardized contrast d ≈ 0.36, *P* < .001). ChatGPT outperformed human responses by an estimated 0.70 points on a 1 to 7 Likert scale.

Question type: No significant main effect of the randomized question was observed, F(4,320) = 0.81, *P* = .522, η^2^ = 0.01.

##### Interaction effects

Author × Question: A significant interaction emerged, F(4,323) = 2.96, *P* = .020, η^2^ = 0.04, showing ChatGPT’s advantage varied by question. The largest effect was for Q5 (d = 0.53, SE = 0.12, *P* < .001; 1.02-point difference), whereas Q2 showed no significant difference (d = 0.03, SE = 0.12, *P* = .82; 0.05-point difference).

Country of Residence: A main effect appeared, F(2,316) = 13.06, *P* < .001, η^2^ = .08. USA participants rated higher than those from Lebanon (β = 0.496, SE = 0.100, *P* < .001; 0.95-point difference) and Switzerland (β = 0.386, SE = 0.089, *P* < .001; 0.74-point difference), whereas Swiss ratings did not differ significantly from the Lebanese group (β = 0.110, SE = 0.088, *P* > .21).

Age was nonsignificant (β = 0.257, SE = 0.149, *P* > .08; ∼0.5-point increase per age group), as was sex (β=-0.028, SE = 0.074, *P* > .70).

##### Sensitivity analysis

The ART ANOVA analysis confirmed these findings.

#### Secondary outcomes

*Comprehensibility*: ChatGPT showed a small but significant advantage, η^2^ = 0.01, d = 0.17 (∼0.27 points), *P* < .01.

*Trust*: ChatGPT rated higher, η^2^ = 0.11, d = 0.34 (∼0.65 points), *P* < .001.

*Reassurance*: ChatGPT rated higher, η^2^ = 0.06, d = 0.26 (∼0.51 points), *P* < .001.

*AI Identification:* No significant difference, η^2^ = 0.00, d = 0.10 (∼0.21 points), *P* > .16. Clear recognition defined as a rating of 1 or 2 for human responses and 6 or 7 for ChatGPT responses was reported in 28% and 33% of cases, respectively (chi-squared test, *P* > .23).

### Qualitative analysis

Of 328 patients, 139 (42%) provided 247 optional comments: 120 on human responses, 95 on ChatGPT responses, and 32 unrelated to the evaluation.

#### Sentiment valence

Comments were coded into 287 meaning units, organized into 4 themes with 7 subthemes and classified by positive or negative valence (Table 4). ChatGPT responses received 124 meaning units: 42 positive (34%) and 82 negatives (66%); whereas Human responses received 163 meaning units: 20 positive (12%) and 143 negatives (88%). Overall, ChatGPT attracted 2.3× more positive comments, whereas human-generated responses received 1.7× more negative comments.

**Table 4:** Qualitative coding framework of patient free-text comments: meaning units categorized by theme, subtheme, and valence with frequency distribution and examples.

Theme	Subtheme	Category	Valence	Human	Chat GPT	Total	Example of Human response related discussion point—patient information	Example of ChatGPT response related discussion point—patient information
1-Comprehensibility& Style	Clarity	Clear	Positive	8	14	22	“The answer was relatively clear and understandable to me” - Male patient, age 40-59, from Lebanon, assigned to question 2.	“I understood what the text said and meant.” - Female patient, age 60-79, from the USA, randomized to question 1.
1-Comprehensibility & Style	Clarity	Unclear	Negative	28	30	58	“Info could have been written more clearly to understand.” - Female patient, age 60-79, from the USA, assigned to question 1.	“It could be simplified into terms that people without medical experience could understand what is being spoken about.” - Female patient, age 18-39, from the USA, assigned to question 5.
1-Comprehensibility & Style	Structure	Poorly structured	Negative	6	4	10	“Structurally and in terms of grammar.” - Female patient, age 18-39, from Lebanon, assigned to question 3.	“By highlighting certain elements, for example by bolding certain elements.” - Female patient, age 18-39, from Switzerland, assigned to question 1.
1-Comprehensibility & Style	Structure	Well structured	Positive	0	1	1	“Well structured.” - Male patient, age 40-59, from Lebanon, assigned to question 1.	“Answer B is more structured.” - Male patient, age 60-79, from Switzerland, assigned to question 2.
2-Information Depth & Rigor	Detail/depth	Adequate	Positive	5	11	16	“It is informative in a different way. It summarizes everything, or more a conclusion.” - Female patient, age 60-79, from the USA, assigned to question 2.	“Very satisfying because it contains details and explains.” - Female patient, age 18-39, from Lebanon, assigned to question 5.
2-Information Depth & Rigor	Detail/depth	Too long	Negative	1	16	17	“Too long.” - Female patient, age 60-79, from Switzerland, assigned to question 2.	“Explanation was quite long.” - Female patient, age 40-59, from Lebanon, assigned to question 1.
2-Information Depth & Rigor	Detail/depth	Too superficial	Negative	57	10	67	“This response is a simplification of a complex topic. (Possibly feeling that, because I just read a much more nuanced and detailed answer from Author A). Would like more detail.” - Male patient, age 60-79, from the USA, assigned to question 5.	“Not very precise.” - Female patient, age 40-59, from Switzerland, assigned to question 4.
2-Information Depth & Rigor	Scientific references	Scientific references absent	Negative	10	8	18	“Give the precise sources and figures.” - Male patient, age 40-59, from Switzerland, assigned to question 3.	“Cite source of statistical info.” - Female patient, age 60-79, from the USA, assigned to question 2.
3-Humanity, Empathy & Trust	Empathy/humanity	Impersonal	Negative	19	6	25	“By putting humanity into the text, which is too cold, and too distant for a patient worried and stressed by the summons and the results of the exam.” - Female patient, age 40-59, from Switzerland, assigned to question 1.	“The human side is missing.” - Female patient, age 18-39, from Switzerland, assigned to question 2.
3-Humanity, Empathy & Trust	Reassurance	Reassuring	Positive	3	4	7	“I thought that author A’s response was ‘consumer friendly’ and technical enough to provide assurance.” - Female patient, age 60-79, from the USA, assigned to question 2.	“Clear, understandable, reassuring.” - Male patient, age 40-59, from Lebanon, assigned to question 2.
3-Humanity, Empathy & Trust	Reassurance	Worrying	Negative	9	8	17	“Not very reassuring.” - Female patient, age 40-59, from Switzerland, assigned to question 4.	“We are certainly more informed, but at the same time we have more anxiety about the risks that may exist.” - Female patient, age 40-59, from Switzerland, assigned to question 1.
3-Humanity, Empathy & Trust	Trust/credibility	Doubtful	Negative	12	0	12	“It’s horrible! Inaccurate, over-generalized, misleading. Doesn’t give advice which patients would want.” - Male patient, age 60-79, from the USA, assigned to question 5.	
3-Humanity, Empathy & Trust	Trust/credibility	Trustworthy	Positive	0	3	3		“Convincing and seems to be based on trustworthy resources.” - Male patient, age 18-39, from Lebanon, assigned to question 3.
4-Global Satisfaction	Global satisfaction	In satisfaction	Negative	1	0	1	“AI-generated response and it does not suit me.”- Female patient, age 60-79, from Switzerland, with high-school education, retired, assigned to question 4.	
4-Global Satisfaction	Global satisfaction	Satisfaction	Positive	4	9	13	“I liked it.” - Male patient, age 40-59, from the USA, assigned to question 1.	“No need to improve, it seems clear to me.” - Male patient, age 40-59, from Switzerland, assigned to question 2.
Total				163	124	287		

Q5 showed the greatest difference in appreciation: Human responses had a valence index of -1.00 (exclusively negative), whereas ChatGPT responses had -0.33 (predominantly negative but mixed). Q2 showed the smallest difference, with Human at -0.33 and ChatGPT at -0.20 (Table S2)

#### Qualitative themes

Clarity (Theme 1: Comprehensibility & Style) was a key area for improvement for both authors. Human responses were sometimes unclear (*n* = 28; eg, “Info could have been written more clearly to understand”) and too superficial (Theme 2: Information Depth & Rigor; *n* = 57; “Would like more detail”). ChatGPT responses were also perceived as unclear (*n* = 30; eg, “It could be simplified for readers without medical experience”) and too long (*n* = 16; eg, “Too long”).

Both authors were criticized for lacking scientific references (Theme 2; *n* = 8 for ChatGPT, and *n* = 10 for Human; eg, “Cite source of statistical info,” and “Give the precise sources and figures”) and were at times worrying (Theme 3: Humanity, Empathy & Trust; *n* = 9 for ChatGPT, and *n* = 8 for Human; eg, “We are more informed, but at the same time have more anxiety about the risks,” and “Not very reassuring”).

Human responses were perceived as impersonal (Theme 3; *n* = 19; eg, “Too cold and distant for a patient worried and stressed”) and doubtful (*n* = 12; eg, “Inaccurate, over-generalized, misleading”). In contrast, ChatGPT received some positive feedback for trustworthiness (Theme 3; *n* = 3; eg, “Convincing and seems to be based on trustworthy resources”) and overall satisfaction (Theme 4: Global Satisfaction; *n* = 9; eg, “No need to improve”).

For reproducibility, a random subset of 46 comments (16%) was independently coded. Inter-rater agreement was high, with Cohen’s κ = 0.86 (z = 22.3, *P* < .001).

### Mixed-methods integration

**Figure 3:**
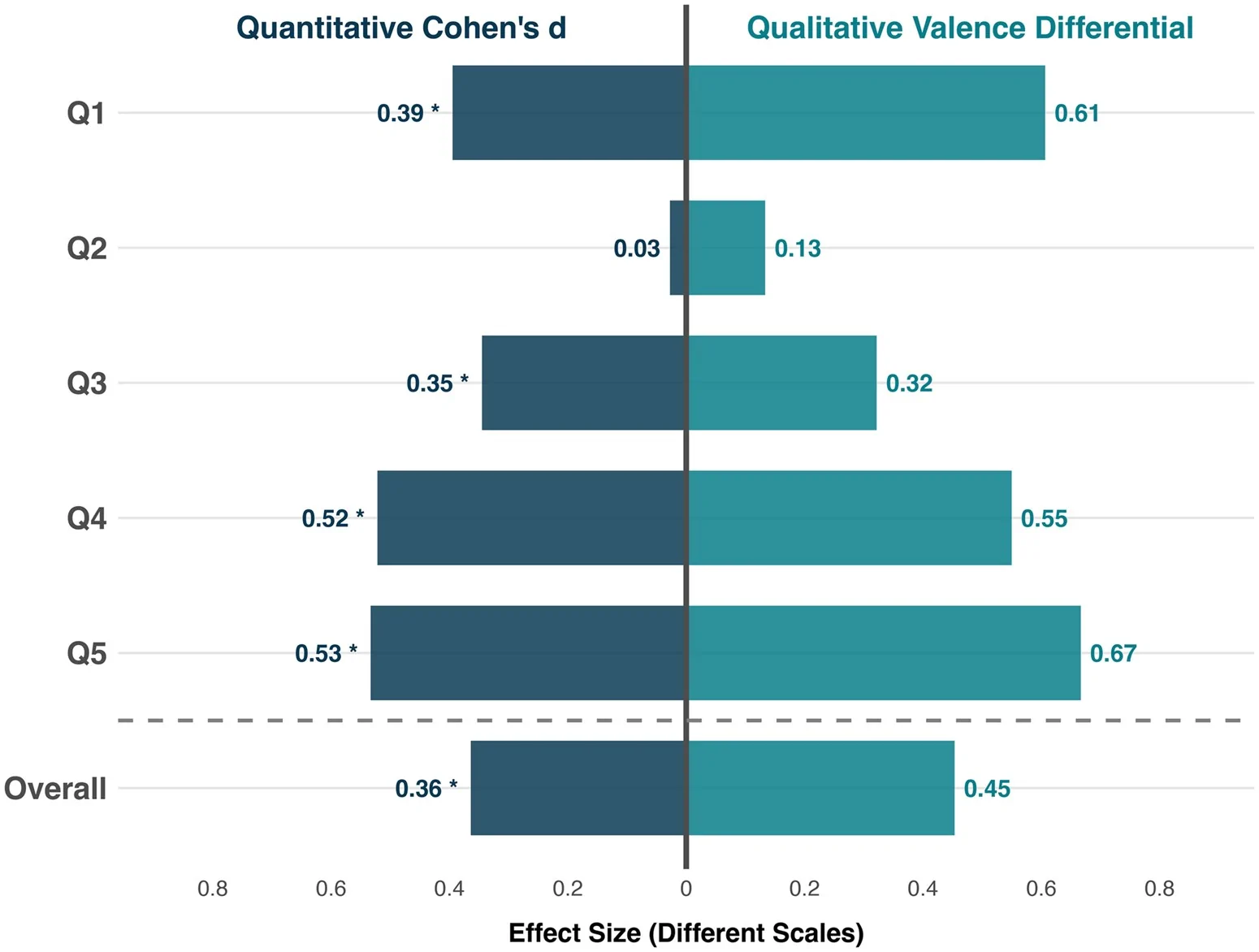
Butterfly chart of quantitative and qualitative ChatGPT advantages over human-generated responses. Both quantitative and qualitative analyses favored ChatGPT over human-generated content, converging in direction and magnitude across all questions. Dark blue bars represent standardized effect sizes (Cohen’s d) derived from the quantitative inverse normal transform (INT) analysis of Likert-scale ratings; asterisks denote *P* < .05 for the INT analysis itself. Turquoise bars represent Valence Differentials from qualitative coding, indicating the directional sentiment of participant comments. For both measures, positive values indicate an advantage for ChatGPT over human responses, whereas negative values would favor human content; however, no negative values were observed, indicating a consistent advantage for ChatGPT across all evaluated questions.

Mixed-methods integration showed convergence between quantitative and qualitative analyses. Author effect sizes from the INT model correlated with Valence Differential scores (*r* = 0.91; 95% CI, 0.15-0.99; *P* < .05). ChatGPT’s quantitative advantage of 0.36 standard effect size (SE = 0.05; 95% CI, 0.26-0.47; *P* < .001) aligned with the qualitative Valence Differential of 0.45 in direction and magnitude across all 5 questions tested (Figure 3).

## Discussion

In this international multicenter study, patients preferred ChatGPT-generated responses over human-written institutional website content across four subjective QUEST domains (satisfaction, comprehensibility, trust, and reassurance). Patients rated ChatGPT responses 0.70 points higher on average on a 7-point Likert scale, with ChatGPT attracting 2.3 times more positive comments and 1.7 times fewer negative comments compared with human-generated institutional content, demonstrating strong alignment between quantitative and qualitative evaluations. Demographic factors such as age and sex had no statistically significant effect, as reported in other studies.^12^^,^^13^ Interestingly, US participants generally rated both authors higher than participants in other countries, suggesting cultural or contextual influences in patient perspective expression.

Beyond patient preference for ChatGPT-generated answers, the free-text comments provide valuable insight into potential patient expectations regarding health information materials. Clarity seems to emerge as the dominant theme spontaneously raised by participants. Some patients reported that institutional websites were overly superficial and lacked sufficient depth. In contrast, they consistently praised ChatGPT for providing more detailed explanations. This assumption is noteworthy given the study context, a potentially stressful and time-constrained setting such as the waiting room prior to an examination. Participants claimed that ChatGPT responses were too long, and in question Q5, the poorest performance of the human-authored response paralleled the markedly greater length of the ChatGPT response, which was nine times longer. However, the length was not perceived as a major drawback. ChatGPT responses being generally longer and more detailed than institutional content may have positively influenced patient satisfaction.

These results contrast with prior expert assessments^4^ reporting ChatGPT-generated materials as overly complex, excessively long, or insufficiently adapted to patients’ needs. These findings, interpreted with the caution warranted by their hypothetical context, may nonetheless challenge prevailing assumptions about how patient-facing materials should be written and simplified.

Unexpectedly, some patients described ChatGPT-generated content as more reassuring and trustworthy. We hypothesize that the empathetic tone and consistent use of polite, supportive language substantially contributed to this perception. This interpretation is supported by question Q2, in which the human-generated response most closely approximated a chatbot empathetic style and was the only item showing no difference in patient preference compared with ChatGPT, in contrast to the other 4 questions.

Notably, patients were equally critical of both ChatGPT-generated and human-authored materials regarding the absence of explicit scientific references. The lack of transparent data sourcing, perceived by some participants as reliance on “black box” algorithms, further underscores the need for greater transparency in medical information delivery.^14^ Importantly, although the clinical accuracy, guideline concordance, or safety of the content provided was not directly assessed in this study, the scientific accuracy of both institutional website content and ChatGPT responses had been evaluated by experts in a prior study.^4^

The observed patient preference for ChatGPT over institutional health professional-written responses raises important concerns. Previous studies have shown that unclear or poorly adapted instructions hinder informed decision-making and information retention.^15–17^ Given that 45% of participants reported prior familiarity with LLMs, and considering their criticism of the institutional materials, there is a risk that patients may increasingly turn to LLMs’ sources of medical information which may generate misleading content owing to unverified data sources, hallucinations, response variability, and unresolved privacy and ethical issues.^18–20^ While offering key explanatory insights, our findings align with previous research demonstrating an overall patient preference for AI-generated written communication and counseling.^21^

Our findings suggest that an expert-guided and clinically validated LLM could represent an effective tool to support radiology risk communication by providing on-demand, personalized explanations. Health professionals should play an active role in the responsible development of such models and in establishing clear regulatory frameworks to mitigate the risk of misinformation. Importantly, involving patients and systematically integrating their feedback into the development of educational materials should be considered essential. Such an approach would better align with patient expectations given that 57% of respondents expressed interest in a dedicated radiology chatbot focused on radiation protection information.

To our knowledge, this is the first international study evaluating AI-generated health information that combines quantitative metrics with qualitative analysis of patient comments. Several limitations should be acknowledged.

First, volunteer-based recruitment may have introduced selection bias toward participants who were more engaged, health-literate, digitally comfortable, or favorably disposed toward technology than the broader radiology patient population. However, the demographic characteristics and the enrollment across 3 culturally distinct countries reflect a broad range of respondent profiles. Moreover, although the content translation from English to French and Arabic-speaking may have influenced interpretation, it ensured consistency with the prior study.^4^ Expanding research to additional languages and clinical contexts is essential.

Second, participants were randomized to questions unrelated to their own scheduled examinations, potentially reducing emotional involvement. Furthermore, participants evaluated a single ChatGPT response without personalization or interaction, and the comparison involved static frequently FAQ content and static ChatGPT-generated responses. Consequently, the findings do not reflect real-world conversational LLM use or interactive FAQ systems. In addition, only 4 institutional websites were included, not widely representative of the online radiology information landscape. However, the objective of this study was to compare patient perceptions of LLM-generated information with verified institutional content rather than to evaluate the online information ecosystem.

Third, the number of questions proposed was limited and selected from a prior expert-based study,^4^ which may not reflect real-world patient information-search behavior. Nevertheless, the questions were retrieved from the FAQ available on official websites that represent typical patients’ concerns. The high completion rate and frequent use of optional free-text comments suggest patient interest and engagement with the topics.

Fourth, we were unable to formally isolate which communication mechanisms (eg, tone/empathy vs structure/clarity/length) drove patient preferences. Moreover, the Author × Question interaction indicates that the magnitude of the ChatGPT-human difference varies across questions, limiting generalizability and highlighting the need to identify determinants of patient preference. Potential order effect may also have been introduced because question order was randomized only in the online format and no washout period was implemented. Moreover, outcomes were restricted to subjective assessments within the QUEST framework. Although the study materials were previously reviewed by 12 international experts, no objective assessment of factual accuracy or concordance with radioprotection guidelines was performed.

Fifth, a temporal mismatch existed between the institutional webpages and LLM outputs. Nevertheless, 4 of the 5 questions remained accessible and unchanged at the time of manuscript submission.

Finally, this study evaluated content generated by a single, older LLM version. Although this choice maximized effect detection in this investigation, model obsolescence is an inherent limitation of all LLM research because these systems evolve continuously. Although newer ChatGPT versions are now available, the findings remain relevant because patient concerns regarding radiation risks are enduring clinical questions. As such, this study provides a useful generational baseline for future comparisons. GPT-3.5's favorable performance may therefore represent a conservative estimate of what subsequent LLM iterations could achieve.^22^

In conclusion, unlike medical imaging experts, patients perceived ChatGPT as superior to human-written institutional websites in radiology risk information. Such a gap between experts and patient perspectives emphasizes the need to improve patient educational materials and suggests that the communication style of LLMs may enhance perceived clarity, understanding, and trustworthiness of medical information. These results provide a starting point for developing patient-oriented educational materials that combine radiology professional expertise with the communication strengths of LLMs.

## Supplementary Material

umag033_Supplementary_Data

## Data Availability

Data generated or analyzed during the study are available from the corresponding author by request.

## Abbreviations

AI: Artificial Intelligence
LLMs: Large Language Models (e.g., ChatGPT)
FAQs: Frequently Asked Questions
QUEST Framework: QUEST Framework
SD: Standard Deviation
IQR: Interquartile Range
AIC: Akaike Information Criterion
INT: Inverse Normal Transformation (based on ranks, used to address non-normality)
ART ANOVA: Aligned Rank Transform Analysis of Variance (non-parametric ANOVA approach)
