# Supplementary material for: Patients prefer ChatGPT to institutional websites for questions on radiation-based imaging exams: international mixed-methods study
Source: Radiol Adv. 2026 Jul 23;3(4):umag033. doi: 10.1093/radadv/umag033 (PMC13495101; doi:10.1093/radadv/umag033)

Supplementary Materials

Patients Prefer ChatGPT to Institutional Websites for Questions on Radiation-based Imaging Exams: International Mixed-Methods Study

Each question was selected from official institutional websites in June 2023, and the original webpage response was treated as human-generated. These webpages were initially chosen in a prior expert-validation study because they were i) readily discoverable using standard Google searches with radiation-protection keywords, and ii) hosted on widely recognized institutional or professional websites, including the International Atomic Energy Agency, Health Physics Society, American Association of Physicists in Medicine, and Yale University, making them plausible proxies for information patients might encounter during routine online searches. Each FAQ and its answer had previously been evaluated for correctness by the expert panel that selected the 12 questions in the prior study. Moreover, the blinded expert assessment reported in that study confirmed the accuracy of the human-generated answers.

ChatGPT-v3.5 responses were also generated in June 2023 using the prompt: “I am a patient, and I have this question” followed by the selected FAQ. The authors attempted to run the same prompt text, using the same model version and default settings in English, across the three participating countries and obtained broadly similar responses from ChatGPT. To avoid straightforward identification of the authorship, authors did not include ChatGPT’s typical expressions of gratitude, and sincere-sounding invitations for continued communication.

For the present study, five questions (Q1–Q5) were selected by consensus among the research team to represent thematic diversity and response format variability (long vs. short, broad vs. precise).

Translation from English into French and Arabic

All the survey content, including the FAQs, the human- and ChatGPT-generated responses as well as the questionnaire, was developed in English. French translation was prepared by a native French-speaking author and reviewed by two additional native French-speaking authors. Arabic translations were produced by a sworn translator and refined by a native Arabic-speaking author for fidelity and Lebanese dialect appropriateness.

Survey Response Settings

Between December 2024 and March 2025, adult patients (≥18 years old) capable of providing informed consent and able to read were consecutively invited to participate while present in the radiology waiting rooms of three institutions (Lebanon, Switzerland, USA). Recruitment was conducted by clinical staff (radiologists, radiographers, medical physicists, receptionists) or via self-enrolment through QR-coded posters displayed in waiting areas. The survey took approximately 15 minutes to complete. Informed consent was obtained orally from all participants prior to participation.

Qualitative Outcomes Analysis:

A hybrid inductive-deductive content analysis was employed to allow themes to emerge from the data (inductive phase) while ensuring structured alignment with predefined Likert scale outcomes (deductive phase) for mixed-methods integration

First, a sample of free-text responses was segmented into *meaning units* (discrete passages conveying a single idea). They were inductively coded by two reviewers (X and X) using descriptive, data-derived labels (e.g., 'unclear terminology,' 'lacks empathy'). These codes were iteratively grouped into broader themes and subthemes, incorporating both positive and negative valences where applicable. Inductive themes were then deductively mapped to the study's predefined outcome domains (Satisfaction, Comprehensibility, Trust, Reassurance). For example, the comment 'unclear terminology' was mapped under 'Clarity: Unclear,' whereas 'very comprehensive response' was mapped under 'Clarity: Clear.' Second, all free-text responses were coded using the finalized mapping framework by one author (X). Third, to ensure reliability, a third author (X) independently coded a random subset of responses using the predefined mapping framework. Inter-rater agreement between (X) and (X) was assessed with Cohen's kappa and discrepancies were resolved through team discussion.

Supplementary Results:

To further contextualize Figure 2, a top-box analysis dichotomizing scores at the ceiling (7/7 vs. <7) revealed a significantly higher proportion of maximal satisfaction ratings for ChatGPT-generated responses compared to institutional website responses (49% vs. 34%; $\chi^2=14.5$, df=1, $p<.001$, 95% CI [0.07, 0.23]).

Table S1: Overall medians, interquartile ranges (IQR), means, standard deviations, minimum and maximum scores for human and ChatGPT-generated responses to patients' questions, presented for each country.

**Author**		**ChatGPT**	**ChatGPT**	**ChatGPT**	**ChatGPT**	**ChatGPT**	**Human**	**Human**	**Human**	**Human**	**Human**
**Outcome**		Satisfaction	Comprehensibility	Trust	Reassurance	AI Identification	Satisfaction	Comprehensibility	Trust	Reassurance	AI Identification
**Lebanon**	Mean ±SD	5.1 ± 2.0	5.3 ± 2.0	4.7 ± 1.8	4.8 ± 1.9	4.3 ± 2.2	4.5 ± 2.1	5.3 ± 2.0	4.1 ± 2.1	4.4 ± 2.0	3.8 ± 2.3
	Median (IQR)	6.0 (3.0)	6.0 (3.0)	5.0 (3.0)	5.0 (2.8)	4.0 (4.0)	4.5 (4.0)	6.0 (3.0)	4.0 (4.0)	4.0 (3.0)	3.0 (5.0)
	Range [min;max]	[1; 7]	[1; 7]	[1; 7]	[1; 7]	[1; 7]	[1; 7]	[1; 7]	[1; 7]	[1; 7]	[1; 7]
**Switzerland**	Mean ±SD	5.5 ± 1.7	5.9 ± 1.5	5.3 ± 1.7	5.0 ± 1.9	4.1 ± 2.2	4.6 ± 1.9	5.6 ± 1.6	4.4 ± 1.9	4.4 ± 1.9	4.0 ± 2.2
	Median (IQR)	6.0 (3.0)	7.0 (2.0)	6.0 (3.0)	5.0 (3.0)	4.0 (4.0)	4.0 (3.0)	6.0 (3.0)	4.0 (3.0)	4.0 (3.0)	4.0 (4.0)
	Range [min;max]	[1; 7]	[2; 7]	[1; 7]	[1; 7]	[1; 7]	[1; 7]	[1; 7]	[1; 7]	[1; 7]	[1; 7]
**USA**	Mean ±SD	6.5 ± 1.0	6.6 ± 0.9	6.0 ± 1.5	5.9 ± 1.5	4.2 ± 2.1	5.4 ± 2.1	6.5 ± 1.2	5.2 ± 2.0	5.1 ± 2.1	4.0 ± 2.0
	Median (IQR)	7.0 (1.0)	7.0 (0.0)	7.0 (2.0)	7.0 (2.0)	4.0 (3.0)	7.0 (3.0)	7.0 (0.0)	6.0 (3.0)	6.0 (3.0)	4.0 (2.0)
	Range [min;max]	[2; 7]	[3; 7]	[1; 7]	[1; 7]	[1; 7]	[1; 7]	[1; 7]	[1; 7]	[1; 7]	[1; 7]

Table S2. Qualitative Sentiment Analysis: Distribution of Meaning Units, Valence Indices, and Valence Differentials Between ChatGPT and Human Responses by Question

This table summarizes the distribution of positive and negative meaning units n_{positive} and n_{negative} extracted from participants' optional free-text comments, directed toward ChatGPT and human-generated responses.

The **Valence Index** was calculated as:

$$\text{Valence Index} = \frac{n_{\text{positive}} - n_{\text{negative}}}{n_{\text{positive}} + n_{\text{negative}}}$$

yielding values between -1.0 (exclusively negative) and +1.0 (exclusively positive sentiment).

ChatGPT's comparative advantage was quantified using the **Valence Differential**:

$$\text{Valence Differential} = \text{Valence Index}_{\text{ChatGPT}} - \text{Valence Index}_{\text{Human}}$$

Positive values indicate more favourable sentiment toward ChatGPT responses.

**Question**	ChatGPT positive meaning units	Human positive meaning units	ChatGPT negative meaning units	Human negative meaning units	ChatGPT Valence Indices	Human Valence Indices	Valence Differential
**Q1**	12	3	18	28	-0.20	-0.81	0.61
**Q2**	10	12	15	24	-0.20	-0.33	0.13
**Q3**	6	3	17	27	-0.48	-0.80	0.32
**Q4**	7	1	16	33	-0.39	-0.94	0.55
**Q5**	8	0	16	31	-0.33	-1.00	0.67
**Overall**	43	19	82	143	-0.31	-0.76	0.45

Figure S1. Example of survey form for question Q5

Evaluation of Radioprotection Responses by Patients

Thank you for evaluating responses to a patient radioprotection question provided by two different authors.

You will review two answers, randomly labeled as Answer A and Answer B, each representing a response from Author A and Author B.

This evaluation is entirely subjective, with no right or wrong answers.

Best regards,
LM-CLEAR team.

LM-CLEAR is an international collaboration between Lausanne University Hospital (Switzerland), Duke University Hospital (USA) and Lebanese University Hospital HLG (Lebanon) institutions that are joining forces to assess if information provided by generative Artificial Intelligence chatbots are robust and reliable compared to information provided by healthcare institutions. For more information, you can contact sofyan.jankowski@chuv.ch

--

Abreviations used in the answers:

AI (Artificial Intelligence): Technology that enables computers and machines to simulate human learning, comprehension and other tasks requiring intelligence.
Chatbot: AI system designed to simulate conversation with users.
LM-CLEAR : Large Model-Communication Language Evaluation About Radioprotection
CT (Computed Tomography): Imaging technique using X-rays to create detailed body images.
MRI (Magnetic Resonance Imaging): Imaging technique using magnetic fields to create detailed body images.

1. The country from which you are filling out the form
   - o USA
   - o Lebanon
   - o Switzerland

2. Your age range
   - o 18-39
   - o 40-59
   - o 60-79
   - o 80 and more

3. Your sex at birth
   - o Male
   - o Female
   - o Prefer not to answer

4. Your educational attainment
   - o Primary school
   - o Middle school
   - o High school
   - o Post high school
   - o Graduate / post graduate

5. Your occupation
   - o Student
   - o Unemployed
   - o Employed
   - o Freelance
   - o Housewife/househusband
   - o Retired

6. The approximate number of imaging test (Ultrasound, Radiographs, CT scans, MRI) you had **in the last 12 months**
   - o 0
   - o 1-2
   - o 3-5
   - o 6-10
   - o More than 10

7. The approximate number of imaging test (Ultrasound, Radiographs, CT scans, MRI) you had **in your life**
   - o 0
   - o 1-2
   - o 3-5
   - o 6-10
   - o More than 10

Evaluation of the answers

In this section, you are invited to read a patient's question along with two responses, one from Author A and one from Author B. You will rate your agreement with several statements, first for Author A's text and then for Author B's text. Please note that **1 represents "No"** and **7 represents "Yes."** The intermediate numbers provide more nuanced options, so choose the one that best reflects your opinion.

If radiation is harmful to humans, how can it also be used to treat cancer?

Response from author A:
Radiation can indeed be harmful to humans, but it can also be used as an effective treatment for cancer. The key to its successful use lies in carefully controlling the dose and targeting the radiation to specific areas of the body where cancer cells are present.

Radiation therapy, also known as radiotherapy, is a medical treatment that uses high doses of radiation to kill or damage cancer cells. It works by damaging the DNA inside the cancer cells, preventing them from growing and dividing further. While radiation can also affect normal healthy cells, the goal of radiation therapy is to maximize the damage to cancer cells while minimizing harm to healthy tissues.

Here's how radiation therapy achieves this balance:

Precise targeting: Modern radiation therapy techniques, such as Intensity-Modulated Radiation Therapy (IMRT) and Image-Guided Radiation Therapy (IGRT), allow for highly accurate and precise targeting of the tumour. This reduces exposure to surrounding healthy tissues, which helps minimize damage to non-cancerous cells.

Fractionation: Radiation treatment is usually delivered in multiple sessions or fractions over several days or weeks. By dividing the total radiation dose into fractions, the healthy cells have time to repair themselves between treatments, while cancer cells are less likely to recover.

Shielding and blocking: Special shields and blocks may be used during radiation therapy to protect nearby organs and tissues that are sensitive to radiation.

Advanced planning and imaging: Prior to treatment, detailed planning is done using advanced imaging techniques to determine the exact location and size of the tumour, as well as nearby critical structures. This enables the medical team to design a treatment plan that delivers the radiation precisely to the tumour while sparing healthy tissues.

Radiosensitivity of cancer cells: Cancer cells are generally more sensitive to radiation than normal cells, making them more susceptible to damage and death at the same

radiation dose that may only cause temporary effects on healthy cells.

While radiation therapy is a valuable tool in cancer treatment, it does have potential side effects, which can vary depending on the area being treated and the overall health of the patient. Common side effects include fatigue, skin changes, and mild inflammation of the treated area. However, most side effects are temporary and resolve after treatment is completed.

It's crucial for patients considering radiation therapy to discuss the potential risks and benefits with their oncologist to make informed decisions about their cancer treatment plan. Every patient's situation is unique, and the decision to use radiation therapy will depend on factors such as cancer type, stage, location, and the patient's overall health.

Mark only one oval per row.

	1 (No)	2	3	4	5	6	7 (Yes)
**The text is understandable**							
**I am satisfied with the answer**							
**This answer is reassuring**							
**The answer was generated from AI**							
**I trust the information presented in the text**							

If radiation is harmful to humans, how can it also be used to treat cancer?

Response from author B:

Radiation can damage cells in the body. Healthy, normal cells are capable of repairing themselves following exposure to radiation. However, cancerous cells have lost this ability. When radiation is delivered to a part of the body containing a tumour, the cancerous cells are killed but the non-cancerous cells survive.

Mark only one oval per row.

	1 (No)	2	3	4	5	6	7 (Yes)
**The text is understandable**							
**I am satisfied with the answer**							
**This answer is reassuring**							
**The answer was generated from AI**							
**I trust the information presented in the text**							

Optional feedback about the two responses
10. How could Author A's response be improved?
Any additional comment? *(optional)*

11. How could Author B's response be improved?
Any additional comment? *(optional)*

Radiation protection and internet

12. Have you ever used AI chatbots (i.e.: ChatGPT) for seeking medical information?

- o Yes
- o No
- o No, but I use it for other purposes
- o I don't know what it is

13. Have you ever sought information on radiation exposure, safety, and potential harmful effects of radiation in medical imaging?

- o Yes, I asked healthcare professionals
- o Yes, on the internet
- o Yes, both
- o No

14. Are you interested in a dedicated chatbot to ask for specific radiology questions including radiation exposure, safety, and potential harmful effects of radiation in medical imaging?

- o Yes, in the radiology waiting room
- o Yes, on the internet
- o Yes, both
- o No

15. Do you have additional questions about radiation exposure, safety, and potential harmful effects of radiation in medical imaging? *(optional)*

The questionnaire is complete—thank you for your participation!

For more information, you can contact sofyan.jankowski@chuv.ch

Figure S2. Violin and Boxplots of Participant Likert Ratings for the Primary Outcome

This figure displays participants' Likert scale scores (1 = "No" to 7 = "Yes") after reviewing one of five patient-question responses generated by humans or by ChatGPT. Participants rated the statement, "I am satisfied with the answer."

The boxplot shows the median (central black bar) and interquartile range (box limits).

The violin plot illustrates the overall distribution of responses.

Grey dots indicate individual observations, with discrete scores ranging from 1 to 7.

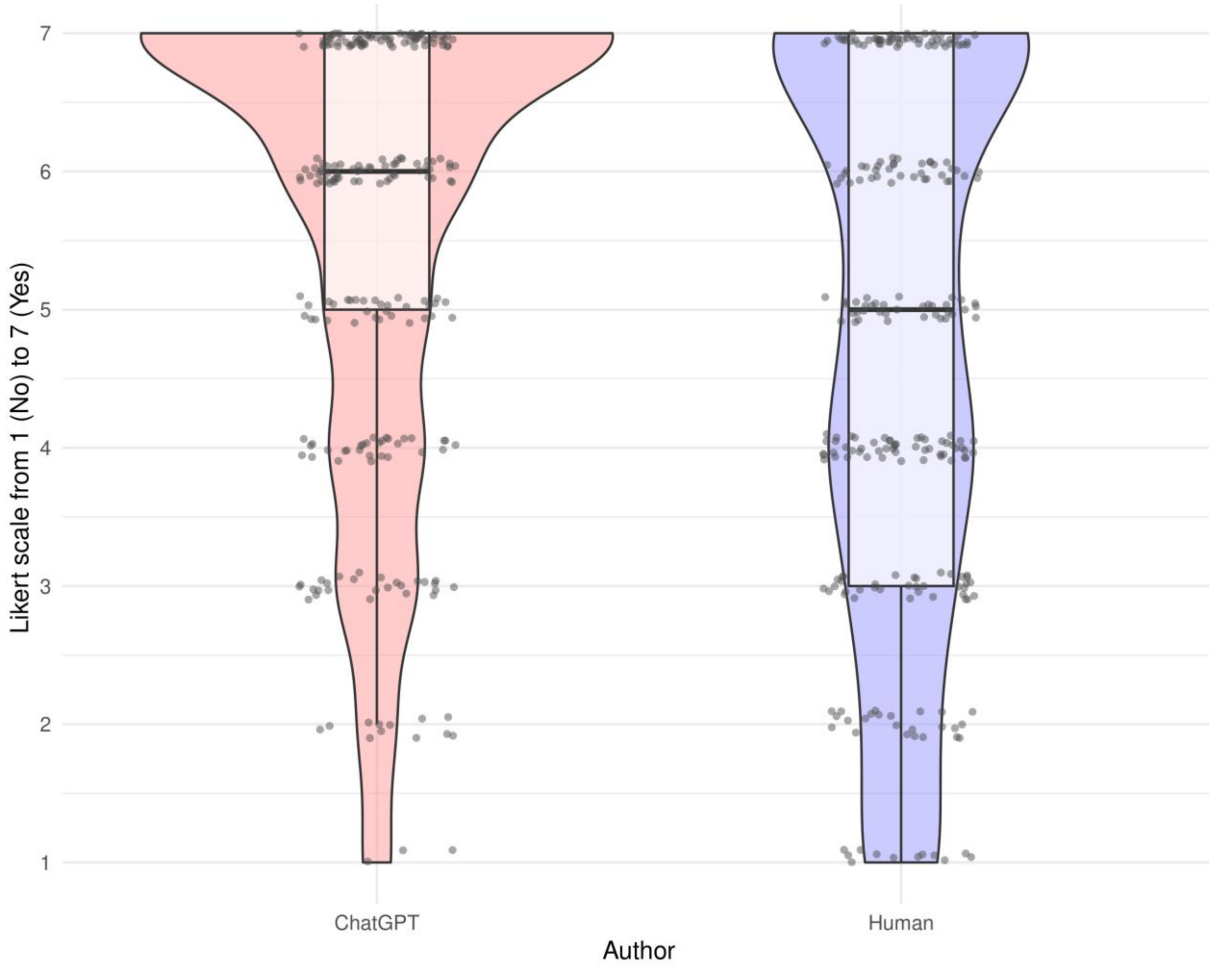

Figure S3. Sankey Diagram Showing Flow of Participant Ratings from Optional Written Comments

A total of 139 patients provided 247 optional written comments. The diagram depicts flows between comment sentiment (central bar: positive vs. negative) and patients' quantitative Likert scores for the primary outcome, "satisfaction" (1 = "No" to 7 = "Yes"). Scores are shown separately for ChatGPT-generated responses (left) and human-generated responses (right). Most positive comments were provided by satisfied patients and were more frequently associated with ChatGPT-generated responses.

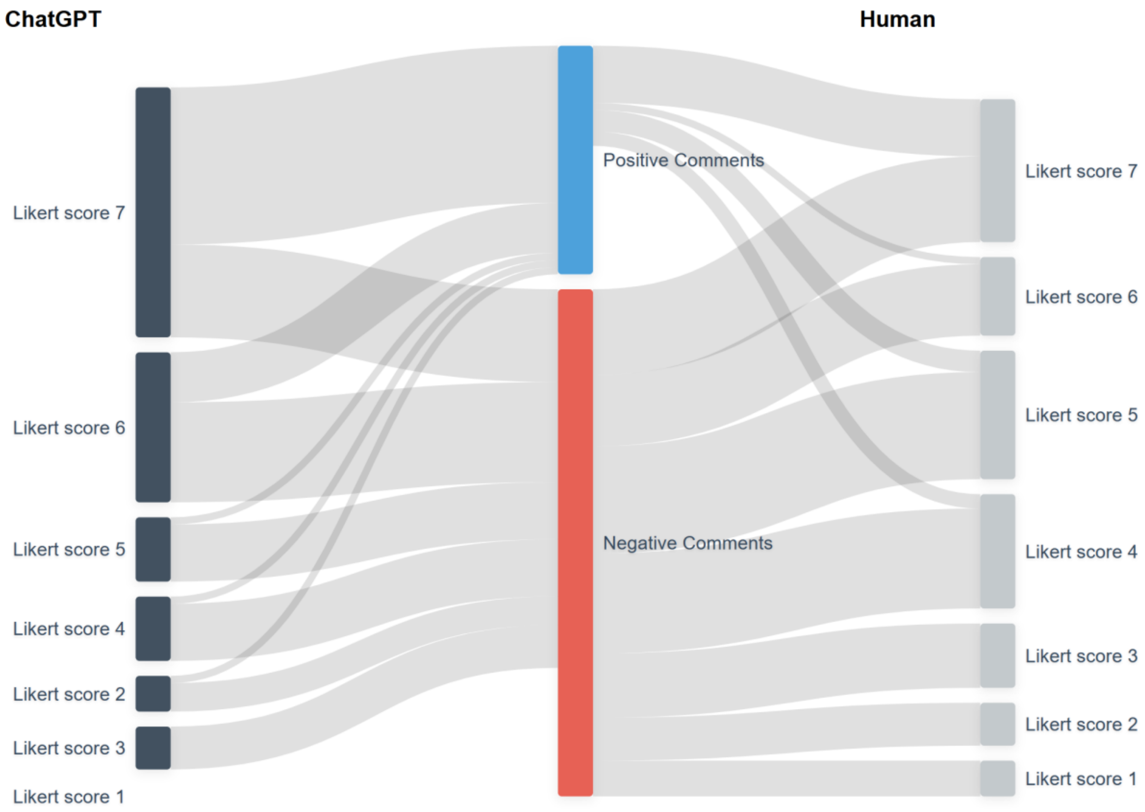

Supplement: Supplementary file 1 [file umag033_supplementary_data.zip › supplemental_materials_final.pdf]
